# Bionomics and insecticides resistance profiling of malaria vectors at a selected site for experimental hut trials in central Cameroon

**DOI:** 10.1186/s12936-018-2467-2

**Published:** 2018-08-30

**Authors:** Benjamin D. Menze, Murielle J. Wondji, William Tchapga, Micareme Tchoupo, Jacob M. Riveron, Charles S. Wondji

**Affiliations:** 1 Vector Biology Department, Liverpool School of Tropical Medicine, Pembroke Place, Liverpool, L3 5QA UK; 2 LSTM Research Unit at the Centre for Research in Infectious Diseases (CRID), P.O. Box 13591, Yaoundé, Cameroon

**Keywords:** Malaria vectors, Insecticide resistance, Characterization, Cameroon

## Abstract

**Background:**

Malaria vectors are increasingly developing resistance to insecticides across Africa. The impact of such resistance on the continued effectiveness of insecticide-based interventions remains unclear due to poor characterization of vector populations. This study reports the characterization of malaria vectors at Mibellon, a selected site in Cameroon for experimental hut study, including species composition, *Plasmodium* infection rate, resistance profiles and mechanisms.

**Methods:**

Indoor resting blood-fed *Anopheles* mosquitoes were collected from houses at Mibellon in 2017 and forced to lay eggs to generate F_1_ adult mosquitoes. Insecticides susceptibility bioassays were performed on the F_1_ adult mosquitoes following the WHO protocol to assess resistance profile to insecticides. The molecular basis of resistance and *Plasmodium* infection rate were investigated using TaqMan genotyping.

**Results:**

*Anopheles funestus* sensu stricto (s.s.) was predominant in Mibellon (80%) followed by *Anopheles gambiae* s.s. (20%). High levels of resistance to pyrethroids and organochlorides were observed for both species. Moderate resistance was observed against bendiocarb (carbamate) in both species, but relatively higher in *An. gambiae* s.s. In contrast, full susceptibility was recorded for the organophosphate malathion. The PBO synergist assays with permethrin and deltamethrin revealed a significant recovery of the susceptibility in *Anopheles funestus* s.s. population (48.8 to 98.1% mortality and 38.3 to 96.5% mortality, respectively). The DDT/pyrethroid 119F-GSTe2 resistant allele (28.1%) and the dieldrin 296S-RDL resistant (9.7%) were detected in *An. funestus* s.s. The high pyrethroid/DDT resistance in *An. gambiae* correlated with the high frequency of 1014F knockdown resistance allele (63.9%). The 1014S-kdr allele was detected at low frequency (1.97%). The *Plasmodium* infection rate was 20% in *An. gambiae,* whereas *An. funestus* exhibited an oocyst rate of 15 and 5% for the sporozoite rate.

**Conclusion:**

These results highlight the increasing spread of insecticide resistance and the challenges that control programmes face to maintain the continued effectiveness of insecticide-based interventions.

## Background

Despite the recent progress made in reducing malaria burden in sub-Sahara Africa through insecticide-based interventions, such as long-lasting insecticidal nets (LLINs) and indoor residual spraying (IRS), malaria remains an important health issue [1]. Malaria control in Cameroon has recently witnessed a significant scale-up of vector control intervention through the distribution of LLINs, pushing up the bed net coverage to 70.9% of the population [2, 3]. However, the effectiveness of these insecticide-based control tools is threatened by the emergence of insecticide resistance in major malaria vectors across African countries, including Cameroon [4, 5]. There is, therefore, a need to design and implement suitable resistance management strategies to preserve the efficacy of existing tools. Use of experimental huts is one of the best approaches employed to assess the impact of resistance on the efficacy of control intervention, such as LLINs and IRS [6–8]. However, monitoring the efficacy of these insecticide-based interventions requires a good characterization of the vectors present in the areas under control, importantly, their species composition, insecticides resistance profiles and resistance mechanisms.

In Cameroon, the main malaria vector species are members of the *Anopheles gambiae* sensu lato (s.l.) complex and the *Anopheles funestus* s.l. group [5, 9–13]. However, these species are not evenly distributed [14–16] and their insecticide resistance profiles vary nationwide [5, 14, 17–20]. Therefore, it is important to characterize local populations in order to assess the impact of vector control interventions.

This study provides a thorough characterization of mosquito vector populations in an area selected for experimental hut trials in Cameroon. The site is located in a geographical transition between forested south and savannah north. This study presents a full characterization of the main malaria vectors in Mibellon, including their species diversity, susceptibility profiles and investigates the molecular basis of the resistance and their *Plasmodium* infection rate.

## Methods

### Study sites

Adult *Anopheles* mosquitoes were collected from Mibellon (6°46′N, 11°70′E), a village in Cameroon located in the Adamawa region, Mayo Banyo Division and Bankim Sub-division. The Adamawa region is in the mountainous area forming a transition between Cameroon’s forested south and savanna north. Mibellon is located in close proximity to permanent water bodies, including a lake and swamps which provide suitable breeding sites for mosquitoes. Human activities are mainly fishing, hunting and subsistence farming, including maize, watermelon and coffee plantations. A survey in the area revealed a high usage of insecticides in the coffee and watermelon farms. These insecticides are mainly pyrethroids, neonicotinoids and carbamates.

### Mosquito collection and rearing

The blood-fed *Anopheles* mosquitoes were collected every morning between 06:00 and 11:00 in the houses. Verbal consent was given by the household owners before starting mosquito collection. Mosquitoes were collected in January 2017 using the Prokopack electrical aspirator (John W Hook, Gainesville, FL, USA) and kept in netted paper cups that were stored in a cool box. Samples were later transported to the insectary of the LSTM Research Unit in Yaoundé (Cameroon).

Mosquitoes were kept 4–5 days until they became fully gravid and then induced to lay eggs using the forced eggs-laying method [21]. The eggs were put in paper cup containing water for them to hatch. After hatching the larvae were placed in trays and reared to adult mosquitoes [21, 22].

### Species identification

The field-caught females were sorted according to morphological keys as previously described [23]. After genomic DNA extraction using the Livak method [24], PCR species identification was performed as described [25] to identify *An. funestus* group members. Whereas the SINE PCR [26] was used to differentiate members of the *An. gambiae* s.l. complex.

### *Plasmodium falciparum* infection rate

Sixty collected *An. funestus* females were cut in two parts: the head and thorax together and the abdomen separately. Genomic DNA was extracted separately from head/thorax and abdomen and the oocyst and the sporozoite rate evaluated using the TaqMan assay [27] using MX 3005 (Agilent, Santa Clara, CA, USA). For *An. gambiae*, due to low number of mosquitoes, the whole mosquito was used and only the overall *Plasmodium* infection rate estimated. One µl of DNA sample was used as template in a 3-step PCR programme with a denaturation at 95 °C for 10 min followed by 40 cycles of 15 s at 95 °C and 1 min at 60 °C. The primers (Falcip + : TCT-GAA-TAC-GAA-TGT-C, OVM + : CTG-AAT-ACA-AAT-GCC, Plas-F: GCT-TAG-TTA-CGA-TTA-ATA-GGA-GTA-GCT-TG, Plas r: GAA-AAT-CTA-AGA-ATT-TCA-CCT-CTG-ACA) were used together with two probes labelled with fluorophores: FAM to detect *Plasmodium falciparum*, and HEX to detect *Plasmodium ovale*, *Plasmodium vivax* and *Plasmodium malariae. Plasmodium falciparum* samples and a mix of *P. ovale*, *P. vivax* and *P. malariae* were used as positive controls.

### Insecticide susceptibility assays

Following WHO protocol [28], F_1_ *An. funestus* sensu stricto (s.s.) and *An. gambiae* s.s. mosquitoes aged between 2 and 5 days were exposed to pyrethroids: permethrin (0.75%), deltamethrin (0.05%) and etofenprox (0.05%), the organochlorine: DDT (4%), the organophosphate: malathion (5%) and the carbamates: bendiocarb (0.1%) and propoxur (0.1%). For each test, mosquitoes exposed to untreated papers were used as control. The assay was carried out at temperatures of 25 °C ± 2 °C and 80% ± 10% relative humidity.

### PBO synergist assays

To investigate the possible involvement of cytochrome P450 s in the observed resistance, female *An. funestus* s.s. were pre-exposed to 4% piperonyl butoxide (PBO) for 1 h and immediately exposed to DDT (4%), permethrin (0.75%) and deltamethrin (0.05%) for 60 min. The mortality was assessed after 24 h and compared with mortality obtained for mosquitoes not pre-exposed to PBO.

### Cone assays

Cone assays were performed using five types of LLINs (Olyset Plus, Olyset Net, Yorkool, PermaNet 2.0 and PermaNet 3.0 (side). These tests were carried out using pieces of LLINs (30 cm × 30 cm). Ten unfed mosquitoes (*An. funestus* s.s. and *An. gambiae* s.s.) females aged 2–5 days were introduced into each cone placed on the LLIN for 3 min. After exposure, the mosquitoes were removed from the cones using a mouth aspirator and then transferred into paper cups and fed with 10% sugar solution. Numbers of mosquitoes knocked-down were recorded after 60 min. A negative control (untreated net) was included in each of the LLIN cone test. Post-exposure mortality was recorded after 24 h of observation. The assay was carried out at temperature of 25 °C ± 2 °C and 80% ± 10% relative humidity.

### Genotyping of L119F-GSTe2: metabolic resistance marker in *Anopheles funestus* s.s.

The L119F-GSTe2 mutation was genotyped to assess the role played by the glutathione S-transferase gene in the DDT and pyrethroid resistance observed at Mibellon as described previously [29]. An allele specific PCR (Tchouakui et al., unpublished) was used to detect the 3 genotypes of the L119F-GSTe2 mutation. Amplification was carried out using PCR parameters of 95 °C for 5 min; 30 cycles of 94 °C for 30 s, 58 °C for 30 s, 72 °C for 45 s, final extension at 72 °C for 10 min. The primers employed for the genotyping are: L119F-Fwd: ATG ACC AAG CTA GTT CTG TAC ACG CT; L119F-Rev: TTC CTC CTT TTT ACG ATT TCG AAC T; L119F-Res1: CGG GAA TGT CCG ATT TTC CGT AGA A**t**AA; L119-F-Sus1: CAT TTC TTA TTC TCA TTT ACA GGA GCG TAaT**C.**

### Genotyping of A296S-RDL in *Anopheles funestus* s.s.

TaqMan genotyping assays were performed in 10 μl volume containing 1 × Sensimix (Bioline), 80 × primer/probe mix and 1 μl genomic DNA. The probes were labelled with two distinct fluorophores FAM and HEX, FAM to detect the resistant allele and HEX to detect the susceptible allele. The assay was performed on an Agilent MX3005 real-time PCR machine with cycling conditions of 95 °C for 10 min, followed by 40 cycles at 95 °C for 15 s and 60 °C for 1 min as described previously [30].

### L1014F and L1014S *kdr* genotyping in *Anopheles gambiae*

The L1014F-kdr mutation and the L1014S-kdr responsible for DDT and pyrethroid resistance in *An. gambiae* s.l. were genotyped in Mibellon mosquitoes. The reaction mixture of 10 μl final volume containing 1 × Sensimix (Bioline), 80 × primer/probe mix and 1 μl template DNA was used for this assay. The probes were labelled with two distinct fluorophores: FAM to detect the resistant allele and HEX to detect the susceptible allele. The assay was performed on an Agilent MX3005 real-time PCR machine with cycling conditions of 95 °C for 10 min, followed by 40 cycles at 95 °C for 15 s and 60 °C for 1 min as previously described [31].

### G119S *ace*-*1* genotyping in *Anopheles gambiae*

The G119S *ace*-*1* responsible to organophosphate and carbamate resistance in *An. gambiae* s.l. was genotyped in Mibellon mosquitoes. Ten μl volume containing 1 × Sensimix (Bioline), 80 × primer/probe mix and 1 μl template DNA. Probes were labelled with two specific fluorophores FAM and HEX, FAM to detect the resistant allele, HEX to detect the susceptible allele. The assay was performed on an Agilent MX3005 real-time PCR machine with cycling conditions of 95 °C for 10 min, followed by 40 cycles at 95 °C for 15 s and 60 °C for 1 min.

## Results

### Field collection

A total of 722 adult female *Anopheles* mosquitoes were collected indoor at Mibellon out of which 584 (80.1%) were morphologically identified as *An. funestus* group and the remaining 138 (19.9%) identified to be *An. gambiae* complex.

### Species diversity among *Anopheles funestus* group and *Anopheles gambiae* complex

Out of the 120 *An. funestus* s.l. mosquitoes randomly selected and tested, *An. funestus* s.s. was found to be the only member of the group present at Mibellon. For *An. gambiae* s.l., out of 89 samples analysed all of them were found to be *An. gambiae* s.s. (S form).

### Insecticide susceptibility bioassays with samples from Mibellon

#### Insecticide susceptibility bioassays in *An. funestus* s.s.

**Fig. 1:**
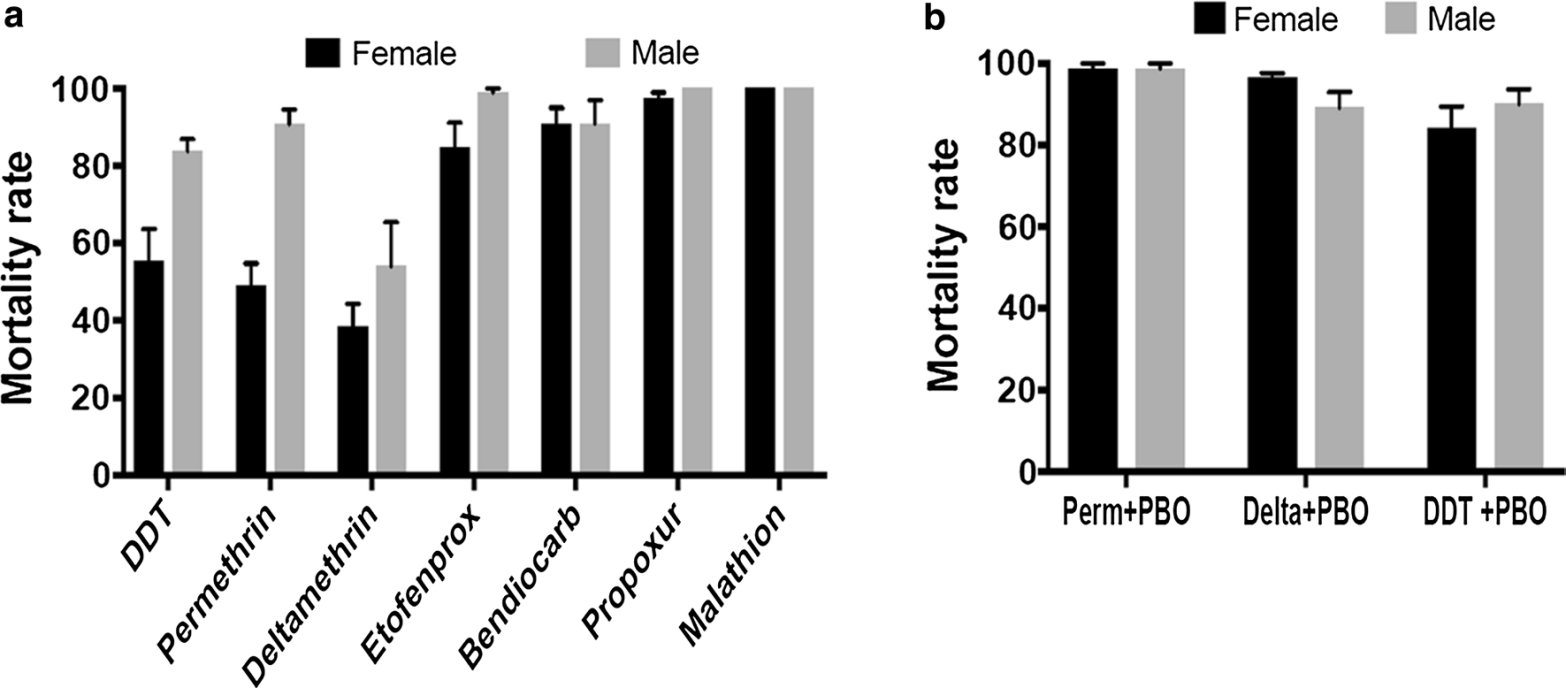
Susceptibility profile of *Anopheles funestus* to insecticides. **a** Recorded mortalities following 60-min exposure of *An. funestus* s.s. from Mibellon to different insecticides. Data are shown as mean ± SEM. **b** Activities of PBO combined to permethrin, deltamethrin and DDT on *An. funestus* s.s. from Mibellon. Data are shown as mean ± SEM

A total of 2700 F_1_ mosquitoes were tested to assess the resistance profile to seven insecticides (Fig. 1). *Anopheles funestus* s.s. (females and males) were resistant to type I and type II pyrethroids. For permethrin (Type I), mortality was 48.88 ± 5.76% for females and 90.72 ± 3.77% for males. For deltamethrin (Type II), mortality was 38.34 ± 5.79% for females and 53.96 ± 11.37% for males. However, mortality was higher for the pseudo-pyrethroid etofenprox with mortality rate of 82.9 ± 8.7% for females and 97.83 ± 2.17% for males (Fig. 1a). For the organochlorine DDT, resistance was observed with mortality rate of 55.28 ± 8.28% for females and 83.78 ± 3.13% for males (Fig. 1a). For the carbamates, a possible resistance was observed against bendiocarb with 90.67 ± 4.3% mortality for females and 95.06 ± 1.97% for males (Fig. 1a) whereas a susceptibility was observed for propoxur with 98.41 ± 1.59% mortality for females and 100% mortality for males (Fig. 1a). Full susceptibility was observed for the organophosphate malathion (Fig. 1a).

#### Insecticide susceptibility bioassays in *An. gambiae* s.s.

**Fig. 2:**
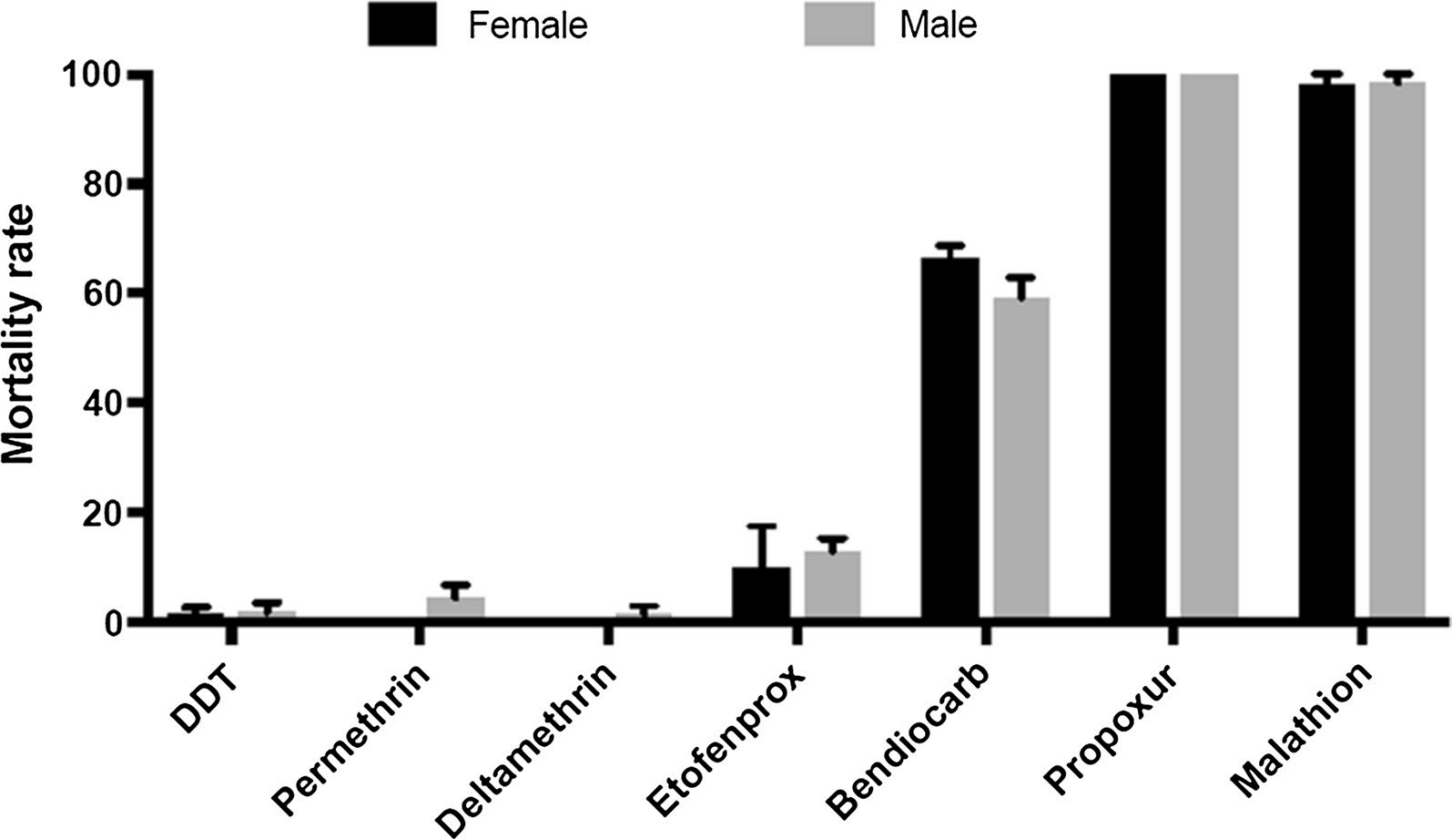
Susceptibility profile of *Anopheles gambiae* to insecticides. Recorded mortalities following 60-min exposure of *An. gambiae* s.s. Mibellon to different families of insecticide. Data are shown as mean ± SEM

A total of 971 *An. gambiae* s.s. were tested to assess the resistance profile to seven insecticides. The *An. gambiae* s.s. population was highly resistant to type I and type II pyrethroids (Fig. 2). For permethrin, mortality was 0 ± 0% for females and 4.44 ± 2.42% for males (Fig. 2). Very high level of resistance was also observed with deltamethrin with no mortality for females and 1.52 ± 1.52% for males (Fig. 2). Resistance was also observed for the pseudo-pyrethroid etofenprox with mortality rate of 9.92 ± 7.66% for females and 13.46 ± 3.93% for males (Fig. 2). High level of resistance to the organochlorine DDT was observed with mortality rate of 1.39 ± 1.39% for females and 1.85 ± 1.85% for males (Fig. 2). For the carbamate bendiocarb, mortality rates of 66.23 ± 2.6% for females and 59.2 ± 3.73% for males were recorded, however, full susceptibility was observed for propoxur (Fig. 2). For the organophosphate malathion, a near full susceptibility was observed with mortality rate of 98.25 ± 1.75% for females and 98.48 ± 1.52% for males (Fig. 2).

#### PBO synergist assays with *An. funestus* s.s.

Pre-exposure of *An. funestus* s.s. to PBO led to recovery of the susceptibility to both pyrethroids type I and II with an increased mortality observed after PBO exposure from 48.88 ± 5.76% to 98.81 ± 3.77% for permethrin and from 38.34 ± 5.79% to 96.54 ± 1.16% mortality for deltamethrin (Fig. 1b). Partial recovery was observed for DDT with increased susceptibility from 55.28 ± 8.28% to 84.16 ± 5.37% mortality after exposure to PBO (Fig. 1b).

### Susceptibility profile against bed nets by cone assays

#### Insecticide susceptibility with cone assays in *An. funestus* s.s.

**Fig. 3:**
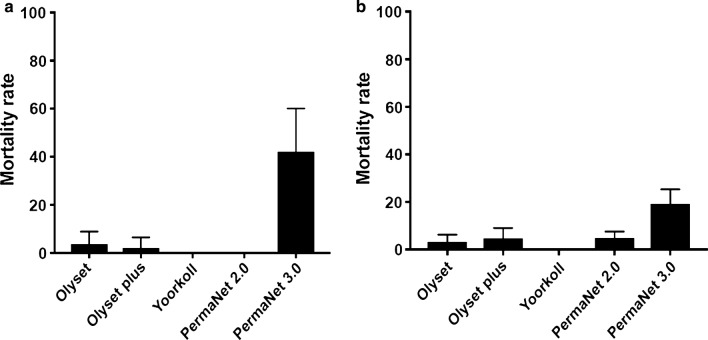
Cone assays with various nets for *Anopheles funestus* and *Anopheles gambiae.* **a** Recorded mortalities following 3-min exposure by cone assay of *An. funestus* s.s. **b** *Anopheles gambiae* s.s. from Mibellon to Olyset, Olyset Plus, Yorkool, PermaNet 2.0 and PermaNet 3.0 (side). Data are shown as mean ± SEM

Low mortality rates were recorded against most of the nets tested. The mortality rates were 2.78 ± 2.78%, and 2.5 ± 2.5%, for Olyset Net and Olyset Plus, respectively; 0 ± 0% and 48.33 ± 6.49% for PermaNet 2.0 and PermaNet 3.0, respectively; and, 0 ± 0% for Yorkool (Fig. 3a).

#### Insecticide susceptibility cone assays in *An. gambiae* s.s.

Similarly, *An. gambiae s.s* showed low mortality rates with the bed nets tested. The mortality rates were 3.13 ± 3.13% and 4.55 ± 4.55% for Olyset Net and Olyset Plus, respectively; 4.77 ± 2.76% and 19.09 ± 6.22% for PermaNet 2.0 and PermaNet 3.0, respectively; and, 0 ± 0% mortality for Yorkool (Fig. 3b).

### Molecular basis of the insecticide resistance in *An. funestus* s.s. population

#### L119F-GSTe2 detection in *An. funestus* s.s.

From 110 F_0_ females collected from the field 7 were homozygous resistant (RR) (6.3%), 48 were heterozygous (RS) (43.2%) and 55 were homozygous susceptible (SS) (49.5%). Overall, the frequency of the 119F resistant allele was 28 and 72% for the L119 susceptible allele.

#### RDL-A296S detection in *An. funestus* s.s.

Out of the 92 samples genotyped, 3 were RR (3.2%), 12 were RS (13.1%) and 75 were SS (83.7%) with frequency of resistant allele of only 9.7% and susceptible allele of 90.3%.

### Molecular basis of insecticide resistance in *An. gambiae* s.s.

#### L1014F/L1014S *kdr* detection in *An. gambiae* s.s.

Out of 72 samples genotyped, 22 were RR (30.5%) 48 were RS (66.6%) and 2 were SS (2.7%) with frequency of the resistant allele 1014F of 63.9% and the susceptible L1014 allele of 36.1%. Out of the 76 samples genotyped for the L1014S marker, 3 were RS (3.9%) and 73 were SS (96.1%), thus a very low frequency of 1.97% was recorded for the resistance allele.

#### G119S ace-1 detection in *An. gambiae* s.s.

Out of 50 samples genotyped, neither RR nor RS were detected as all the samples were found to be SS.

### *Plasmodium* infection rate in the *An. s funestus* s.s. population in Mibellon

**Fig. 4:**
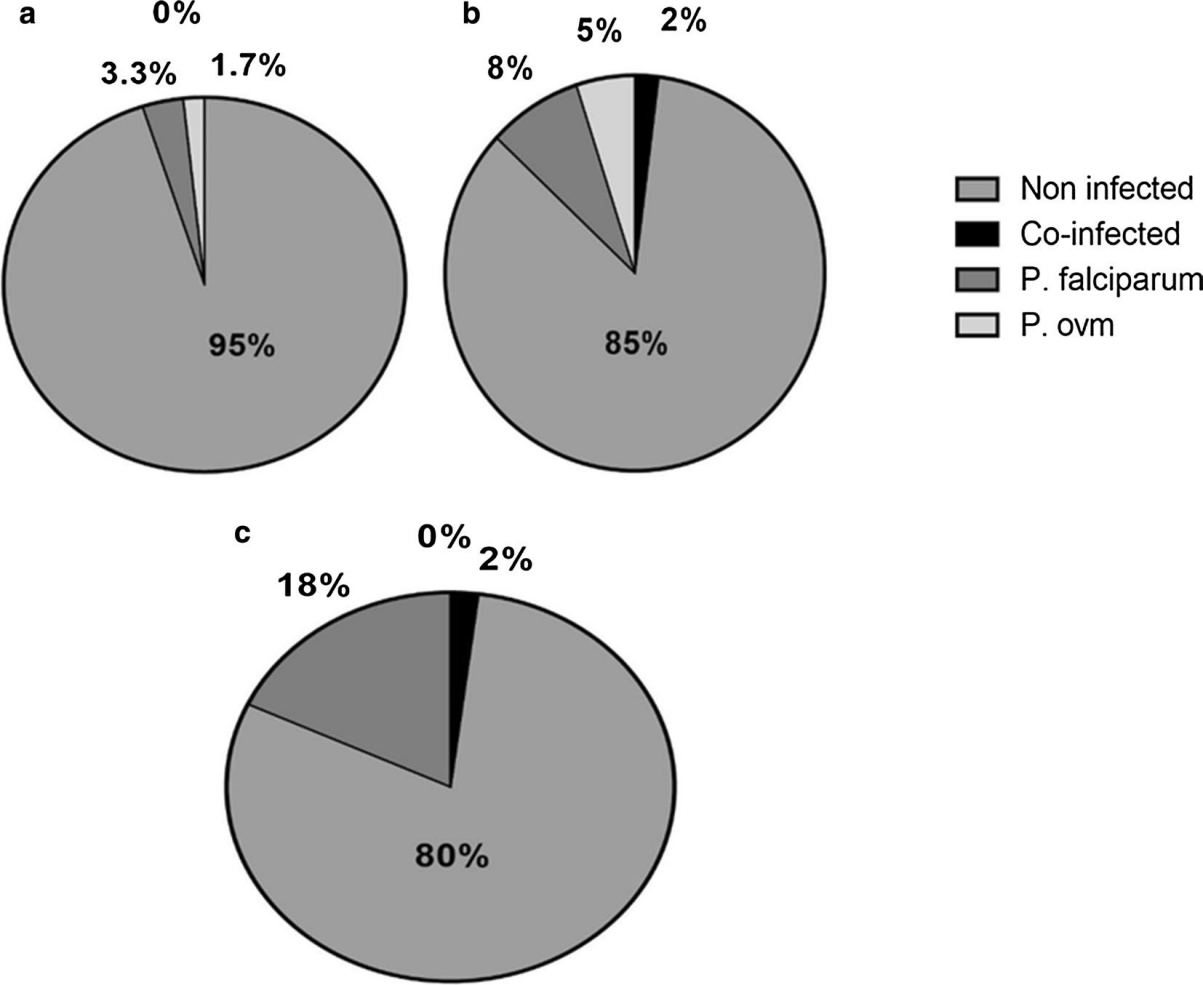
*Plasmodium* infection rate in malaria vectors from Mibellon in 2017. **a** *Plasmodium* infection rate in *An. funestus* s.s. with head and thorax. **b** *Plasmodium* infection rate in *An. funestus* s.s. with abdomen only. (C) *Plasmodium* infection rate in *An. gambiae* s.s. for whole mosquitoes

A total of 60 *An. funestus* from Mibellon were tested for *Plasmodium* infection using TaqMan from the head-thorax and from the abdomen separately. The analysis of the head and thorax revealed 3 (5%) mosquitoes infected, which included 2 (3.3%) *P. falciparum* and 1 (1.7%) infection which is either *P. ovale*, *P. vivax* or *P. malariae.* However, 8 (15%) mosquitoes were detected infected when the abdomen was examined, including 5 (8.3%) *P. falciparum*, 3 (5%) infections which are either *P. ovale*, *P. vivax* or *P. malariae* and 1 (1.7%) mixed infections (Fig. 4).

### *Plasmodium* infection rate in the *An. gambiae* s.s. population in Mibellon

A total of 60 *An. gambiae* were tested for *Plasmodium* infection from the whole body. The analysis revealed 12 (20%) mosquitoes infected, which included 11 (18.3%) *P. falciparum* and 1 (1.7%) mixed infection (Fig. 4).

## Discussion

Characterization of malaria vector populations is a prerequisite for the implementation of successful vector control interventions and for assessing the impact of insecticides resistance. This study comprehensively characterized the main malaria vectors in Mibellon, a location in Cameroon chosen for the implementation of experimental huts to assess the efficacy of insecticide-based interventions.

### Species composition

Out of the 9 species of *An. funestus* group described, only *An. funestus* s.s. was detected in Mibellon. This result is similar to the one observed in the northern part of Cameroon, in Gounougou, where *An. funestus* s.s. was 99.5% of the total collection and *Anopheles leesoni* was 0.5% [5]. The predominance of *An. funestus* s.s. in the *An. funestus* group is also mentioned in West Africa as observed at Kpome in Benin [30]. The focus on indoor blood-fed mosquito collection in this study may have prevented to collect the outdoor resting members of the group. Nevertheless, this is in contrast with the distribution of members of this group observed in eastern and southern parts of Africa where several members are collected indoors. For instance, in Uganda *Anopheles parensis* was found predominantly indoors [32, 33]. Similarly, many other species of the *An. funestus* group have been collected indoors in southern Africa, including *An. parensis, An. leesoni* and *Anopheles rivulorum* [34–36]. For the *An. gambiae* complex*, An. gambiae* s.s., previously known as S form, was the only species found. This result is similar to previous observations showing that *An. gambiae* s.s. was the main species in localities south of the Adamawa mountains, characterized by humid savannah and forested areas [10, 37].

### The multiple insecticide resistance in both major vectors is a challenge for vector control

The multiple resistance pattern observed in this *An.s funestus* population is similar to the pattern observed in the northern part of the country, with the exception of bendiocarb resistance which was higher in the north [5]. This result suggests that resistance in *An. funestus* is widespread in Cameroon and could be a concern for effective insecticide-based control tools against this vector. *Anopheles funestus* is highly resistant to both type I and II pyrethroids. The resistance observed in Mibellon seems higher than in Gounougou (2012). This difference could be due to the length of time between the two studies as resistance could have increased since 2012 when the study at Gounougou was carried out. It could also be evidence that the pyrethroid resistance level in *An. funestus* in Cameroon is increasing. This increase could be due to the massive distribution of LLINs implemented by Cameroonian Government in the past 5 years. Mibellon is also located in an area where agriculture is the main activity, and agricultural use of pesticides could be another factor that is driving the increase in resistance level. A similar level of increase of pyrethroid resistance was observed in southern Malawi [35] and also reported in Uganda for *An. funestus* [32]. The high recovery of susceptibility observed after PBO exposure for deltamethrin, permethrin and DDT suggests that cytochrome P450 genes are playing a significant role in these resistance patterns. Altogether, this resistance to pyrethroids should be of great concern for malaria control programmes in Cameroon. If no strong action is taken to manage it, there is a risk that the massive distribution of pyrethroid-impregnated LLINs taking place in Cameroon could be jeopardized. The possible resistance observed to the carbamate bendiocarb in *An. funestus* population shows that such insecticide should be ruled out as an alternative to pyrethroid for IRS. The full susceptibility to the organophosphate malathion, as also demonstrated across the continent so far, suggests that this insecticide class is the most suitable for IRS against this species.

Very high level of resistance to several classes, including organochlorine, pyrethroid and carbamate, was also observed in *An. gambiae* s.s. population. This high resistance in *An. gambiae* is in line with the increased resistance reported in this species in several sites across Cameroon [14, 17–20]. The resistance in *An. gambiae* was higher than in *An. funestus* for most insecticides (e.g., no mortality against permethrin in *An. gambiae vs* 40.9% in *An. funestus*) suggesting a greater selection operating in *An. gambiae*. This could be explained by a selection of resistance from breeding sites contaminated with insecticides used for the protection of the crops. As *An. gambiae* breeding sites are often located at the vicinity of crops, the selection could be greater in this species compared to *An. funestus* because of mosquito-breeding sites in Mibellon, where there is a large lake that insecticides from farms drain into.

### Bio-efficacy of LLINs in cone assays

The low mortality rates observed with permethrin and deltamethrin for both species in Mibellon correlated with the reduced bio-efficacy of most LLINs observed with cone assays including PBO-based nets such as Olyset Plus. This loss of efficacy may be due to selection pressure induced by the massive distribution of bed nets by the government [2, 3] in addition to the use of pesticide for farming in the area. For both species, the mortality for all the nets is very low except for PermaNet 3.0 (side). For *An. funestus*, the mortality rates for all LLINs are lower than has been recently reported from other countries such, as DR Congo [38]. However, because cone assays could underestimate the efficacy of LLINs, as they do not assess their additional excito-repellent effect [39], future studies with experimental huts are needed to establish the impact of resistance on LLINs in this region.

### Predominance of metabolic resistance in *An. funestus* contrasts with high frequency of knockdown resistance in *An. gambiae*

The near full recovery observed for pyrethroids in *An. funestus* after pre-exposure to PBO indicates that resistance is mainly conferred by metabolic resistance particularly by cytochrome P450s [40]. This is in line with previous reports of the absence of *kdr* in this species in Cameroon [5] and across Africa [41]. The frequency of the 119F-GSTe2-resistant allele in Mibellon field population (28%) is lower than in the northern part of Cameroon in Gounougou (52%) [5] or in Ghana (44.2%) [42] and Benin (56.25%) [30]. The frequencies in Mibellon are closer to that observed in the eastern part of Africa in Uganda (20.4%) [21, 32]. GSTe2 has been shown to confer cross-resistance between DDT and pyrethroids [42]. The partial recovery of DDT susceptibility after PBO assays suggests that GSTe2 is probably playing a role in the resistance in this *An. funestus* s.s. population.

The frequency of the 296S-RDL-resistant allele is only 9.7%, which is lower than was observed in the northern region at Gounougou. This low frequency could be as a result of recovery to susceptibility for dieldrin after this insecticide was removed from public and agricultural sectors in Cameroon, as observed in Gounougou where the frequency of this mutation went down from 80% in 2006 to 40% in 2012 and 14.6% in 2015 [5, 43]. Such reversal of resistance is encouraging for the implementation of resistance management strategies.

The very high resistance levels to pyrethroids in *An. gambiae* s.s. (no mortality to permethrin), correlates with the high frequency of the 1014F *kdr* allele (63.9%). This is in line with other reports from Africa where high pyrethroid resistance in *An. gambiae* s.l. has been associated with nearly fixed *kdr* allele in the population, as observed recently in DR Congo [38], or previously in Côte d’Ivoire [44]. However, this high level of *kdr* is in contrast to frequencies observed in other locations across Cameroon as highlighted by a recent review [17]. The very low frequency of the 1014S *kdr* allele in Mibellon is similar to previous reports across Cameroon showing that this marker, originally present in East Africa, has now migrated to Central and West Africa although still at very low frequencies [45]. Overall, the fact that 1014F *kdr* frequency is not fixed in Mibellon in the presence of such high pyrethroid and DDT resistance suggests that other mechanisms are playing an important role, probably metabolic resistance as shown for other *An. gambiae* s.l. populations in Cameroon [18, 19, 46]. Further investigation of the resistance mechanisms will help elucidate the molecular basis driving resistance in this population. The total absence of the 119S *ace*-*1* mutation is in line with the susceptibility of this population to organophosphate and carbamate as this mutation is responsible for organophosphate and carbamate resistance [47, 48].

### Malaria transmission roles of both vectors

This study further confirms the role of *An. funestus* in malaria transmission with sporozoite infection rate of 5%. This is lower compared to that observed [49] in Nkoteng where *P. falciparum* infection rate was found to be 8.6%. In Benin (Kpome), *An. funestus* population was found with high *Plasmodium* infection during the dry season (infection rate of 18.2%), although from the whole mosquitoes [30]. The higher infection rate found in abdomen (15%) compared to head and thorax (5%) in this study further supports the significant barriers that the midgut plays in preventing oocyst migration to salivary gland [50]. The number of *An. gambiae* s.s. infected with *Plasmodium* (20%) is higher compared to *An. funestus* s.s. (15%). This rate is very high compared to previous results in Cameron (6.5–8.1%) [12]. This could be linked to the higher level of insecticide resistance in the *An. gambiae* population, because they are resistant and live longer, which could increase their ability to be infected.

## Conclusion

Multiple resistance observed in both *An. funestus* s.s. and *An. gambiae* s.s. at Mibellon in central Cameroon is a concern for ongoing insecticide-based interventions although the full susceptibility to organophosphate offers an alternative to IRS. However, the impact of such multiple resistance on the effectiveness of insecticide-based control interventions needs to be evaluated, notably through experimental huts. The presence of resistance in both major vectors makes this area suitable for such studies.

## Abbreviations

ADN: deoxyribonucleic acid
DDT: dichlorodiphenyltrichloroethane
PCR: polymerase chain reaction
LLINs: long-lasting insecticidal nets
IRS: indoor residual spraying
Kdr: knockdown resistance
*s.s*: sensu *stricto* (in the strict sense)
s.l.: sensu lato
PBO: piperonyl butoxide
WHO: World Health Organization
